# Sealing in Arsenic

**Published:** 1904-11

**Authors:** 


Sealing in Arsenic.—Prepare the cavity margins for permanent fillings. If gum hemorrhage ensue pack with cotton saturated with adrenalin chloride while preparing application and filling. When everything is ready remove cotton, syringe cavity with warm water, lay in a piece of asbestos felt, about large enough to cover the floor of the cavity; cover the cervical wall of the cavity with amalgam until the filling reaches beyond gum margin; press the asbestos felt away from the floor of the cavity, and place the application in position. Press back the asbestos felt over the application, and fill the balance of the cavity. When it is desirable to remove the nerve, drill down behind the filling, extending the cavity sufficiently to admit of direct access to the nerve canals, or, if sufficient enamel wall exists between the filling and the point directly over pulp chamber to warrant it, open through the fissures without any regard to the filling. This method protects the gum from action of arsenic, and simplifies the after-treatment.—*Domi. Jour.*

